# Physical Mechanism of Selective Healing of Nanopores in Condensed Matter under the Influence of Laser Irradiation and Plasma

**DOI:** 10.3390/nano14020139

**Published:** 2024-01-08

**Authors:** Zhiqiang Wang, Ivan Vladimirovich Ushakov, Ivan Sergeevich Safronov, Jianping Zuo

**Affiliations:** 1 School of Energy and Mining Engineering, China University of Mining and Technology (Beijing), Beijing 100083, China; wzhiqianglhm@126.com; 2 Physics Department, National University of Science and Technology “MISIS”, 119049 Moscow, Russia; issafronov@yandex.ru; 3 School of Mechanics and Civil Engineering, China University of Mining and Technology (Beijing), Beijing 100083, China

**Keywords:** physics of nanopore healing, strength, nanohardness, extreme conditions, selective laser irradiation

## Abstract

The investigation of the features of laser control over the state of nanoscale objects in solid materials is an urgent task of condensed matter physics. We experimentally established the potential for the simultaneous enhancement of hardness and resistance to surface cracking in a titanium alloy due to selective laser irradiation. The regularities of selective heating near nanopores and the influence of the nanopore system on the features of isotherm propagation have been revealed. A physical model is proposed for the healing of nanopores situated in the surface layer of the sample. According to this model and as a result of laser irradiation and laser plasma, a brief transition of the material surface to extreme conditions is initiated.

## 1. Introduction

The physics of selective interaction of short-pulse laser irradiation with inhomogeneous (defective) areas in condensed matter is a perspective both in the field of condensed matter physics and in the field of physical materials science [1,2,3,4,5,6]. The integral physical properties of some materials are determined by the state of local areas characterized by micro- and nanodimensions. For example, such materials include the optical elements of high-power laser systems. The optical strength and mechanical integrity of these materials are determined by inhomogeneous areas with sizes ranging from 10^−10^ to 10^−6^ m [7,8,9]. Optical breakdown is initiated in micro- and nanoscale defective areas due to irradiation by laser impulse. As a result, the mechanical damage/destruction of the sample takes place. In such cases, the integral exploitation characteristics of the sample are determined by processes in inhomogeneous/defective areas with micro- and nanoscale dimensions.

Traditional methods of thermomechanical processing using laser technologies, as a rule, provide treatment of the entire sample/surface. In this case, the properties and structure of the entire sample are changed [10,11,12]. However, if the integral characteristics of the material are determined by nano- and microareas, effective processing should ideally focus solely on influencing those areas, without effecting the “defect-free” material and without altering its properties.

The first scientific investigations devoted to the selective interaction of laser irradiation with defective areas were made on optically transparent materials. Under certain conditions, laser irradiation may influence defective areas only [7,9]. During selective processing, laser irradiation does not affect the “defect-free” material and does not change its structure and properties. In the article of [7], the specificity of selective laser treatment was experimentally investigated. The possibility to transform the defective areas into nondangerous conditions (for example, due to the relaxation of mechanical stresses) was shown. The transformation of defective areas into nondangerous conditions (in optically transparent materials) during selective laser processing is explained by the following phenomena: the destruction of absorbing inclusions; the relaxation of mechanical stresses; and the partial or complete healing of micro- and nanoscale pores and cracks.

In the subsequent stages of research devoted to investigating the specific characteristics of selective laser treatment, optically opaque materials were used. Multicomponent amorphous and amorphous–nanocrystalline metallic alloys were used. For opaque materials, the selectivity of laser treatment can be realized in a thin surface layer [13].

Currently, there are numerous experimental studies devoted to changing the mechanical properties of metallic and amorphous alloys under the influence of laser irradiation [14,15,16,17]. Laser technologies for processing metals and alloys may differ significantly from traditional thermomechanical processing methods [18,19,20,21,22]. As a result of the selective effect of short-pulse laser irradiation on the defective areas of nanocrystalline metal alloys, the effects of simultaneous increases in microhardness and plastic characteristics were found. In the works of [2,13,14,23], significant increases in the hardness (nano- and microhardness) and plastic characteristics associated with the resistance of the surface to crack formation under intense local loading of the diamond pyramid have been determined. The local increase in the strength characteristics of the material was qualitatively explained on the basis of ideas about the possibility of laser healing of defects such as pores and microcracks.

Of all types of three-dimensional (volumetric) defects, micro- and nanopores are of the greatest interest. An ideal nanoscale pore, as a physical object, has a number of unique physical and mechanical properties. An ideal nanoscale pore has several unique properties, e.g., a spherical shape and an absence of stress concentrators. In addition, heat exchange due to irradiation is actually prohibited in it, which is due to the very small size of the nanopore. Heat exchange due to irradiation can only be realized by high-energy photons with small wavelengths. The thermal conductivity in such a system is also near a zero value due to absence of a heat-conducting substance.

The surface layer of a solid material always has a higher density of defects [24,25]. In the case of amorphous metals/alloys, the surface layer has high density pores (the density of an amorphous metal alloy is always lower due to the excess and structurally determined free volume) [26]. Many nanocrystalline materials obtained by controlled annealing of amorphous metallic alloys can have excessive porosity [1]. In the case of a traditional metal alloy, the surface also has an increased density of defects, including nano- and microsize pores. They are also often defects in materials manufactured using laser additive technology [15,16,17,27,28,29,30,31,32].

Previously, many authors investigated the process of pore healing in solid materials, for example, under the influence of elevated temperatures and pressures [25,26]. Different mechanisms of pore healing may be realized (such as vacancy mechanism; dislocations mechanism; viscous mass transfer; etc.). In [33,34], molecular dynamic modeling of changes in the porosity of the surface layer initiated by ultrashort laser pulses was carried out. The authors showed that, after laser processing, porosity reduction is possible. In the cited investigations, the changes in porosity were considered within the context of processes that affect the structure and properties of the entire material. Alterations in porosity, combined with the material’s structure, resulted in a modification of all the properties.

In this investigation, for the first time, a selective laser processing technique was experimentally used for a titanium alloy, which was previously developed for an amorphous nanocrystalline material. For the first time, the selective influence of laser irradiation on nanopores has been researched using computer simulation. For the first time, the healing of pores using inhomogeneously heated material has been theoretically investigated.

The aim of the investigation is to research the physical regularities of the selective healing of nanopores on the surface layer of a crystalline material as a result of the effects of laser irradiation and plasma.

## 2. Materials and Methods

The investigation into changes in mechanical properties as a result of laser pulse irradiation was made on a titanium alloy (VT18u trademark, the average composition is provided on the website https://scraptraffic.com/splav/vt18u, accessed on 6 March 2023). The surface preparation of the titanium sample involved sanding and polishing before irradiation [23].

Laser processing was carried out using the LS-2136 laser system (supplied by JV “LOTIS TII,” Republic of Belarus, Minsk; equipment details available on the equipment website https://lotis-tii.com, accessed on 21 February 2023). The key technical parameters of the laser system are outlined in Table 1.

**Figure 1:**
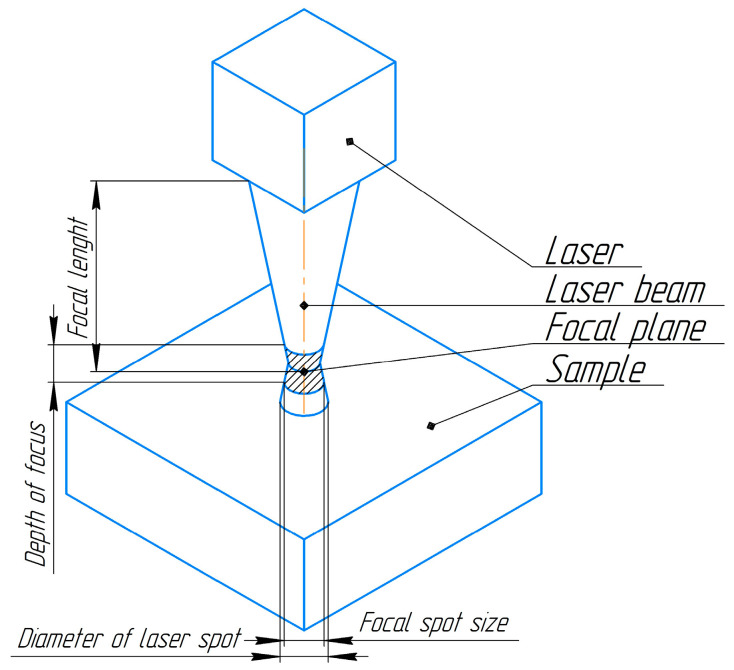
The scheme illustrates the focusing of the laser beam. The focal plane is situated at a height of 1 mm above the surface of the sample.

Focusing was achieved using an optical system with a diaphragm, thus resulting in a laser beam diameter of 100 µm in the focus area. The sample was processed under normal conditions in an air environment. The laser impulse was focused over the treated surface at a height of 1 mm. A schematic representation of the effects of a single-laser irradiation pulse is illustrated in Figure 1. The power density of the laser pulse in contact with the material surface was maintained at 24 ± 0.1 GW/m^2^. Laser exposure to the metal surface is an intricate process. In accordance with articles [35,36], an impulse with energy of 75 mJ induces a gas–plasma cloud. As a result of the influence of the gas–plasma cloud on the material, a shock wave is initiated. Ultrafast heating of the local region takes place. The temperature of the gas–plasma cloud can exceed 3000 K, thereby leading to the melting of the sample surface. Under these conditions, heating and cooling rates can reach 10^−6^–10^−7^ K/s [36].

**Figure 2:**
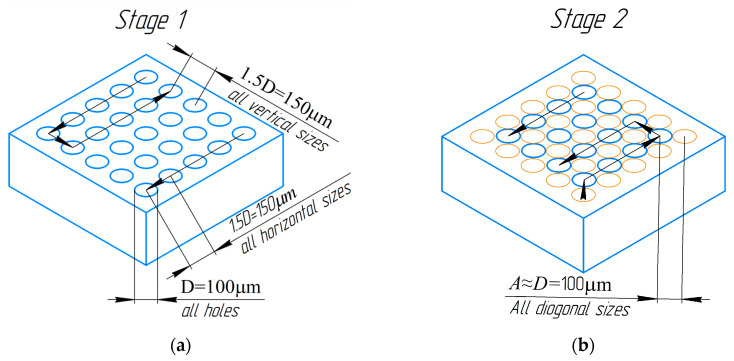

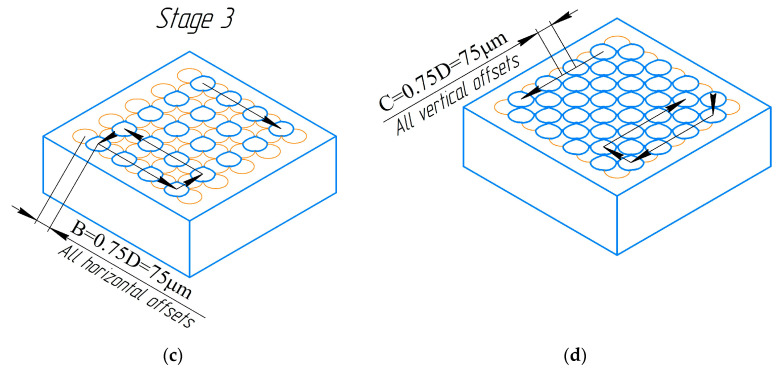
Sample processing scheme. The lines and arrows show the trajectory and direction of movement of the sample during laser processing. Blue circles are current processing stage. Orange circle are previous processing stage. (**a**) The first stage of processing with creation of a matrix of nonoverlapping areas. (**b**) The second stage without overlapping of melted areas. (**c**) The third stage with overlapping of melted areas. (**d**) The final stage with full treatment of all surface.

The positioning system was configured in such a way that the surface of the sample was processed in several stages. The first stage allowed for the creation of a system of circular areas (melted zones). The centers of these circular areas were situated at a distance of 1.5D = 150 ± 2 mkm from each other. The overall scheme of the successive processing stages is illustrated in Figure 2, where lines and arrows depict the processing trajectory. The second stage (without overlapping) was carried out with a shift relative to the last melting zone at a distance of 100 microns diagonally (Figure 2b). This made it possible to process the nonmelted areas between the adjacent four areas. Similar matrices were formed at the third and fourth processing stages, and they were shifted relative to the previous ones (Figure 2c,d). In the third stage, the laser beam was focused with a shift relative to the center of the last melted area at a distance of 75 microns vertically (Figure 2c). The fourth (final) processing stage (with overlapping) was carried out with a shift relative to the lowest zone of the third stage at a distance of 100 mkm diagonally (Figure 2d). The total size of the resulting processing area was 5 × 5 mm. It is noteworthy that, during the experiments, the diameter of the irradiation zone could vary, but the general processing algorithm remained unchanged. The overall temperature increase of the sample during selective laser treatment did not exceed 10 K.

Microphotographs of the treated surface were obtained using an inverted metallographic Olympus BX51 (Olympus Corporation, Tokyo, Japan) microscope. Images of the surface were taken in reflected polarized light (special built-in software programs were used). The observation of the laser processing area was carried out both in the light and dark field mode.

The results of the nanohardness measurement were obtained using the CSM TTX nanoindentor with the NHT2 module and AFM wide scan (CSM Instruments, Needham, USA, website https://smtnet.com/company/index.cfm?fuseaction=view_company&company_id=53901, accessed on 24 February 2023). The main technical characteristics of the equipment include the following: a vertical load range (used range for indentation) of 0.1 to 500 mN; a load resolution of 0.04 micron; a loading speed of up to 10,000 mN/min; a maximum depth of the print of 40 microns; and a depth resolution of 0.004 nm. Nanoindentation was accomplished using the Oliver–Pharr method. The Berkovich diamond pyramid was used with a maximum load of 0.05 N and an approach speed of 8 µm/min.

The microdestructions and microhardness were investigated using PMT-3 installation (METROTEST, Neftekamsk, Russia). The main technical characteristics of the equipment include the following: a limit of test load of 0.0196~4.9 N; a limit of permissible error of loads in the main/additional ranges of no more than ±1.5%; and a limit of absolute error in measuring the length of the diagonal prints of 0.0004 mm.

## 3. Results

### 3.1. Experimental Results

**Figure 3:**
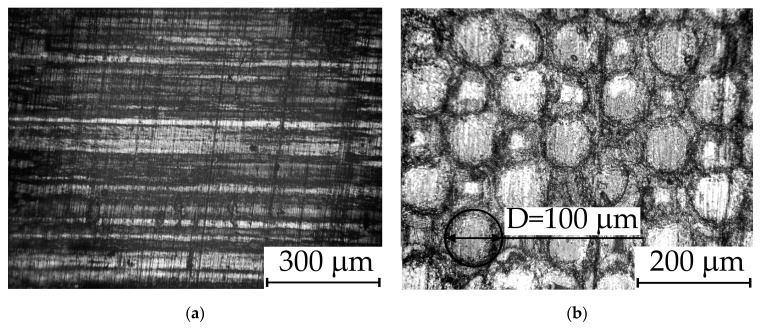
(**a**) Microphotograph of untreated surface. (**b**) Microphotograph of the irradiated surface. Visible areas of laser melting have the shape of circles with a diameter of 100 mkm.

Micrographs of the treated area and untreated one are presented in Figure 3. The shape of the melted areas is a circle with a diameter of 100 ± 5 microns. The processing areas from the previous stages are nearly invisible, and the initial boundaries are significantly distorted due to the overlapping of molten boundaries. The micrograph highlights the area of one of the craters after laser exposure with a dark circle.

**Figure 4:**
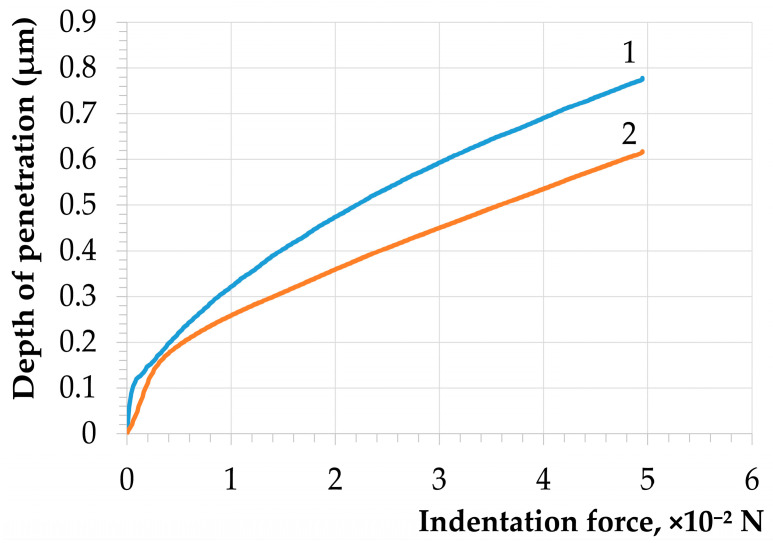
The dependence of the indentation depth on the force: 1—untreated sample; 2—laser-irradiated sample.

Figure 4 shows the dependencies of the load during nanoindentation on the indentation depth.

The results were calculated using the CSM Instruments Indentation Software (Version 3.8.0, TTX-NHT S/N: 01-05230) to measure the nanohardness according to the Oliver–Pharr method [23,37]. The elastic modulus values for the untreated and treated material were 103.9 GPa and 176.84 GPa, respectively (Figure 4).

The nanohardness value for the untreated sample was 4.32 GPa. After selective laser treatment, the nanohardness value was 6.22 GPa. As shown in Figure 4, for curve No. 1, fluctuations in the dependence of the indentation depth on the force can be noted in the indentation depth range from 0.1 to 0.2 µm. These fluctuations indicate the presence of roughness on the material’s surface and three-dimensional defects in the surface layer.

After laser treatment, uneven areas disappeared, and a more even movement of the indenter was observed. In previous works [23], a fourfold increase in nanohardness was found for some titanium alloys after selective laser treatment.

A distinctive feature of the discussed laser processing method is that a significant increase in hardness is accompanied by an increase in plasticity, such as resistance to crack formation under loading conditions determined using a Vickers diamond pyramid. A series of mechanical tests was made where the sample’s surface was indented with a Vickers pyramid (PMT-3), and the statistics of crack initiation due to local loading were determined (Table 2).

If at least one crack was formed as a result of indentation using the Vickers pyramid, it was considered that the loading was accompanied by cracking. The samples were loaded with a force ranging from 0.25 N to 4.9 N. For each force level, 20 loadings were performed, and the average microhardness value was calculated and recorded in the Table 2. For the untreated surface, the mathematical expectation of the probability of crack formation after indentation was 0.2 at F = 0.49 N and 0.55 at F = 4.9 N. There was no surface cracking on the laser-treated surface after local loading using the Vickers pyramid in the range loads of 0.49 N and up to 4.9 N inclusive. Of course, if we were to increase the load on the indenter, then (after exceeding some of the load on the indenter) cracks will be formed. The microhardness grew according to the increase in indentation force (corresponding to an increase in the contact area and according to the theoretical expectations). However, an increase in the load on the indenter to 1.96–4.9 N caused a distortion of the prints, and, for these loads, the microhardness values were indicated in an approximate range.

Brittle destruction of the untreated surface may be caused by the presence of defects in the surface layer. High mechanical stresses near the nano- and microdimensional defects can initiate crack growth. After selective laser treatment and as a result of defects healing, resistance to cracking must be increased.

### 3.2. Theoretical Results

At the current frontier of scientific experimentation equipment, a detailed exploration of the simultaneous impact of high temperatures and shock waves on nanopores in real-time remains unattainable. Consequently, computer modeling is a good tool for the investigation of the heating and deformation of nanopores. Computer simulation was conducted in two stages.

In the first stage, the process of heating (isotherm propagation) of the surface layer was simulated. The surface layer contains groups of nanopores. The regularities of the heating of the material in the vicinity of the nanopores and the influence of the geometry of such a system on the specifics of the propagation of the isotherms were studied. This made it possible to clarify the physical regularities of heating and to begin the second part of the computer simulation. In the second stage, the process of the deformation and healing of a nanopore located in a nonuniformly heated material was simulated.

The effect of irradiation using a single-laser impulse was simulated. Titanium alloy is opaque at a laser wavelength of 532 nm. Laser irradiation leads to rapid heating of the surface, ablation of the material, and the formation of a cloud of laser plasma. After the formation of a gas–plasma cloud, the mechanism of heating is changed. The gas–plasma cloud partially shields the energy of the laser impulse. Further heating of the material using a laser impulse is caused by the effect of a gas–plasma cloud [2,23,35,36].

A two-dimensional model was used. The model was based on rapid heating of the surface of a titanium alloy without taking into account internal heat sources. The revealing of the features of the isotherms distribution was carried out by comparing models of defect-free material and models of material with a pore system. The calculation zone was taken in the form of a rectangle with dimensions of 40 × 11 mkm. Boundary conditions were imposed on the calculation zone. To take into account the ablation of a substance from the surface of a solid material in the first nanoseconds of irradiation, the free boundary of the sample (which was subjected to initial heating) was set in the form of an elliptical form. The major semiaxis of the ellipse was set to a = 10 mkm, and the minor semiaxis was set to b = 1 mkm. To reduce the calculation time, the size of the local heating area (the major semiaxis of the ellipse) was reduced by five times. Such an approach did not affect the accuracy due to the small size of the nanopores and the size of the selected boundary heating region (Figure 3).

A significant change in the mechanical properties after selective laser treatment was observed in a surface layer with a thickness of 0.5–1 mkm [2,13,23]. Consequently, the sizes of the pores and the sizes of the pore system were chosen so that they were located in a layer with a thickness of less than 1 mkm. The configuration and dimensions of the pore system were such that a maximum temperature gradient was realized during heating. The initial temperature of the zone was 293.15 K. Heating was carried out from the free surface of the irradiated zone of the sample. The initial temperature of the heated surface was set close to the melting point of titanium at 1943 K. The initial temperature of the medium was maintained at the vertical boundaries, the lower boundary, and the upper boundary. Boundary conditions of the first kind were used. The surface temperature coincided with the temperature of the material adjacent to the surface.

The main requirement of the physical model of selective laser processing is the ability of laser irradiation (and/or the processes initiated by it) to selectively affect local inhomogeneous/defective areas. Let us consider the thermal conductivity equation [38,39,40,41] from the point of view of the selective laser treatment model (1):

(1) $$\rho C_{p}\frac{\partial T}{\partial t}+\vec{\nabla }∙\left(-k\vec{\nabla }T\right)+\rho C_{p}\vec{u}∙\vec{\nabla }T=Q,$$

where *ρ* is the solid density of titanium alloy (kg/m^3^), *C_p_* is the heat capacity (J/(kg·K)), *Q* is the heat source term (W/m^3^), *u* is a vector-valued convective velocity field (m/s), and *k* is the solid thermal conductivity (W/(m·K)). The *k* value describes the relationship between the heat flux vector $\vec{q}$ and the temperature gradient $\vec{\nabla }$*T*; it is defined according to Fourier’s law of heat conduction, which for a two-dimensional model can be written in the form of Equation (2):

(2) $$\vec{q}=-k\left(\vec{i}\frac{\partial T}{\partial x}+\vec{j}\frac{\partial T}{\partial y}\right),$$

Within the framework of the physical model, the task is nonstationary. Accordingly, the first term of the equation $\frac{\partial T}{\partial x}$ is nonzero. Based on experimental and theoretical data [33,34], depending on the mode of interaction of the short-pulse laser irradiation with the material, both the healing of micro- and nanopores and an increase in porosity are possible. The increase in porosity is associated with such processes like melting, crystallization, and convection. Crystallization (taking into account a decrease in the material volume) may increase porosity. The third augend in Equation (1) takes into account the thermal conductivity in the material in the presence of convection. Therefore, one of the conditions for the selective laser processing of nano- and micropores with minimal changes in the structure and properties of the surrounding material is (1) the third augend in Equation (1) must be zero, or (2) there must be minimal contribution of the third augend to the processes of heating the surface layer of the material. This possibility has been experimentally demonstrated in the works of [2,23] and theoretically established in the work of [34]. For the same reason, the power of the internal heat source is assumed to be zero. In that case, Equation (1) takes the following form (3):

(3) $$\rho C_{p}\frac{\partial T}{\partial t}+\vec{\nabla }\cdot \vec{q}=0,$$

This form of the thermal conductivity equation most accurately represents the physical and mathematical problem at the current level of research, meets the criteria of a scientific investigation, and was used for simulating the thermal/laser effects on a titanium alloy using the finite element method.

## 4. Discussion

In this investigation, we researched the spread of isotherms in an ideal sample (without defects) and the propagation of isotherms in a sample with various configurations of pores: one pore, a horizontal row, a vertical row, and a triangle. The most informative results were obtained using the “triangle” geometry.

**Figure 5:**
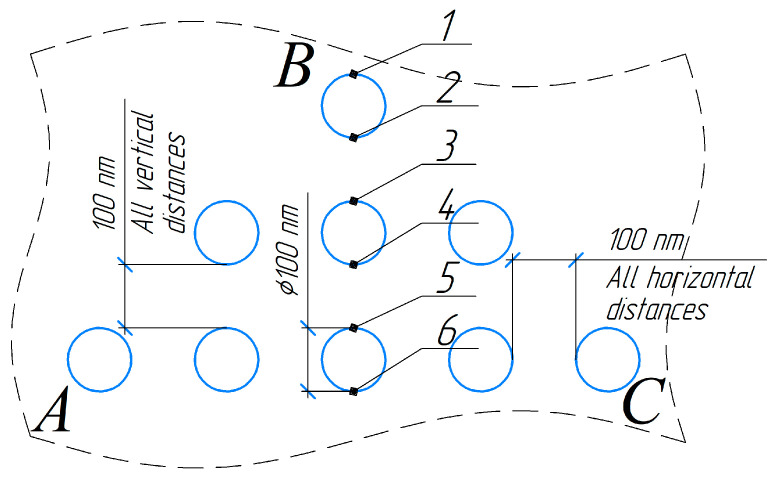
A model of the geometry of the “triangle” pore system. Points 1, 3, 5 are the boundary areas of the material adjacent to the upper part of the pore. Points 2, 4, 6 are the boundary areas of the material adjacent to the lower part of the pore. Horizontal and vertical distances between pore boundaries are 100 nm.

The geometry of the equilateral triangle of pores *ABC* is illustrated in Figure 5. The distance from the treated surface to the upper pore was 100 nm.

As a result of computer simulation using different software programs (FreeCAD Version 0.20, https://www.freecad.org/, accessed on 20 February 2023; Gmsh Version 4.10.3, https://www.freecad.org/?lang=ru, accessed on 20 February 2023; ElmerGUI Version 9.0, https://www.csc.fi/web/elmer, accessed on 20 February 2023; and FEATool Multiphysics Version 1.16, https://www.featool.com/, access on 6 March 2023), it was determined that the material heating before the top of the upper pore always occurred faster than in a defect-free material. Simultaneously, the heating of the material with this pore was slowed down in comparison to the defect-free material.

**Figure 6:**
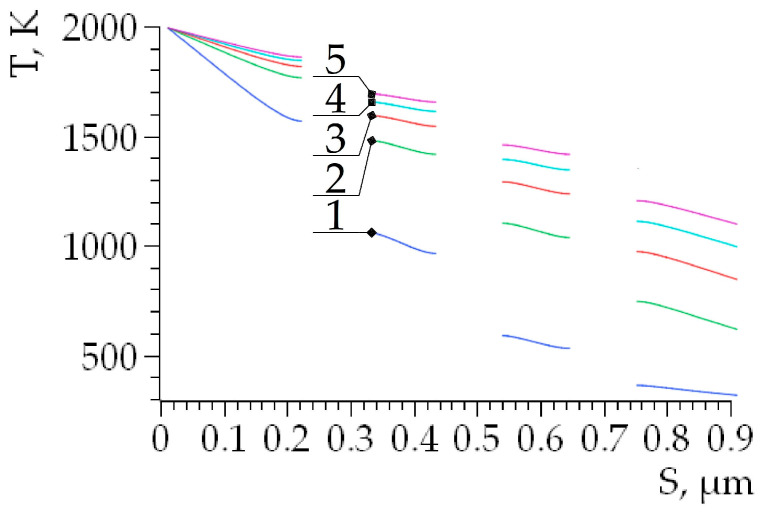
Changes in temperature on a straight line along the height of the triangle *ABC* drawn from the vertex *B* to the base *AC*. *S* is the distance to the sample surface. The discontinuities on the dependency curves correspond to the pores. Curves: No. 1—10^−8^ s; No. 2—3 × 10^−8^ s; No. 3—5 × 10^−8^ s; No. 4—7 × 10^−8^ s; No. 5—9 × 10^−8^ s.

Figure 6 illustrates the specific temperature decrease with increasing distance (*S*) from the sample surface (local heating area) along the height of the triangle *ABC*, which was drawn through vertex *B* to the base. Discontinuities on the graph indicate the location of pores with near-zero thermal conductivity. The temperature difference at the upper and lower boundaries of the pore was able to reach 500 K in the initial stages of heating (Figure 6, curve No. 1).

**Figure 7:**
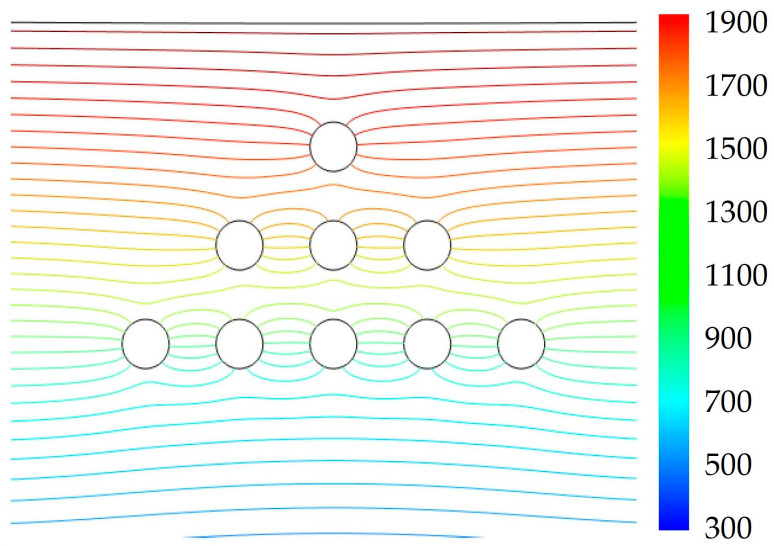
Results of computer simulation of isotherm propagation in a sample.

During the modeling process, we analyzed the distribution of the isotherms over two geometric features of the triangle *ABC* (Figure 5): (1) along the height drawn from vertex *B* to the base *AC* (central part) and (2) along one of the sides (*AB* and *BC*) of the equilateral triangle of pores *ABC* (side face). The simulation revealed that the distribution of isotherms during the heating of the material along the vertically arranged triad of pores (the central part of triangle *ABC*) was symmetrical. The symmetry of the isotherm distribution on the side faces *AB* and *BC* of the triangle *ABC* was slightly distorted (Figure 5 and Figure 7).

According to the simulation results, the relationship between the temperature difference at points above the pores (Figure 5, points 1, 3, and 5) and below the pores (Figure 5, points 2, 4, and 6) was established. The temperature *T*_1_ in the material adjacent to the upper point of the pore (e.g., point 1) and the temperature *T*_2_ at the lower point of the pore (e.g., point 2) were numerically determined. The temperature increment for each pore was determined by the difference between the higher and lower values: $T_{1}=T_{1}-T_{2}$. Similarly, the temperature increments at these points were calculated for the case of the defect-free material: $T_{1}^{/}=T_{1}^{/}-T_{2}^{/}$.

**Figure 8:**
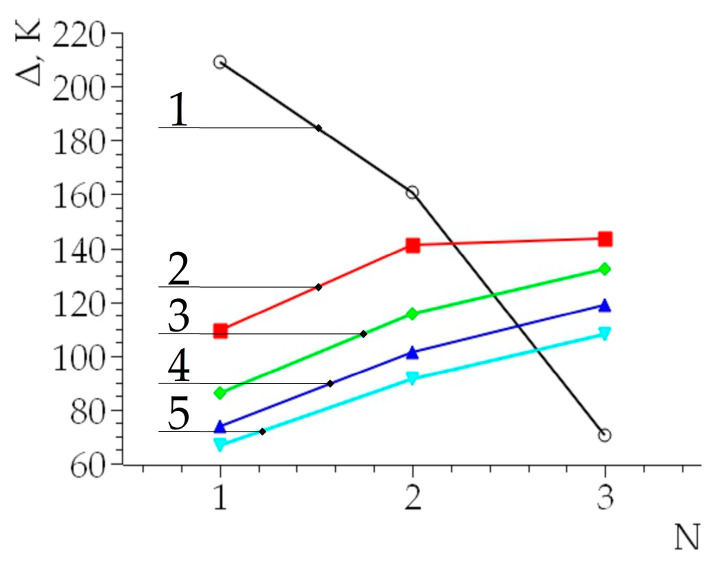
Relative temperature difference *Δ,K* for the first, second, and third pores in the titanium alloy VT18u. N—number of pores from surface of a sample. Curves: No. 1—10^−8^ s; No. 2—3 × 10^−8^ s; No. 3—5 × 10^−8^ s; No. 4—7 × 10^−8^ s; No. 5—9 × 10^−8^ s.

Based on these calculations, the additional contribution of the pores to the difference in the temperature increment ($=T_{1}-T_{1}^{/}$) was determined (Figure 8). The temperature difference Δ was always greater for a material with pores than for a defect-free material (for these conditions, Δ was always greater than zero).

The distribution of isotherms in the vicinity of the pores affects the physics of the process of partial/complete healing of the pores under selective laser irradiation. Before the upper pore, the temperature was significantly higher than in the defect-free material. Under the upper part, the temperature was much lower in comparison to the defect-free area.

Considering that laser exposure is accompanied by the formation of a shock wave with a shock pressure of up to 10^10^ Pa [2,35,36] (these are the standard calculations based on the technical characteristics of the LS-2136 Table 1), conditions were created for the movement of heated material in the direction of the shock wave propagation. The efficiency of the material movement depends on the duration of the laser impulse (laser plasma), the characteristics of the heated material (rheological parameters of the continuous medium), and several other factors. For all the pores shown in Figure 7, the direction of propagation of the compression pulse was vertically downward (perpendicular to the base *AC* of the triangle *ABC*). Consequently, plastic movement of the heated material toward the pore can be expected. Additionally, due to the temperature gradient above and below the pore, deformation and filling with a more heated substance may occur. Considering the proposed model (no gases in the pore), complete healing of the pore is possible, thereby filling the entire space with easily movable components of a metal alloy.

Simulation of the pore healing process under the impact load was made using QForm Version 10.2.4 (http://www.qform3d.ru, accessed 20 February 2023) and FreeCAD Version 0.20 (https://www.freecad.org/, accessed 20 February 2023), thereby taking into account the isotherm distribution shown in Figure 7. Elastic deformation was neglected during modeling, and the problem was solved using the finite element method [40,42]:

(4) $$\left\{\sigma^{\prime }\right\}=\frac{\bar{\sigma }}{\dot{\bar{\varepsilon }}}\left[D\right]\left\{\dot{\varepsilon }\right\},$$

where $\left\{\sigma^{\prime }\right\}$ is the matrix column (vector) of the stress deviator (5); $\bar{\sigma }$ is the effective stress; $\dot{\bar{\varepsilon }}$ is the effective strain rate (intensity of the strain rate); [D] is the coupling matrix (6); and $\left\{\dot{\varepsilon }\right\}$ is the matrix column of the strain rates (7):

(5) $$\left\{\sigma^{\prime }\right\}=\left\{\begin{array}{c} \sigma_{xx}-\sigma_{m}\\ \sigma_{yy}-\sigma_{m}\\ \sigma_{zz}-\sigma_{m}\\ \sigma_{xy}\\ \sigma_{yz}\\ \sigma_{zx} \end{array}\right\},$$

where ${\sigma^{\prime }}_{ij}$ is the stress deviator, and i,j∈x|y|z; $\sigma_{m}$ represents the average stresses.

(6) $$\left[D\right]=\frac{2}{3}\left[\begin{array}{ccc} \begin{array}{ccc} 1 & 0 & 0\\ 0 & 1 & 0\\ 0 & 0 & 1 \end{array} & \begin{array}{ccc} 0 & 0 & 0\\ 0 & 0 & 0\\ 0 & 0 & 0 \end{array} & \begin{array}{ccc} 0 & 0 & 0\\ 0 & 0 & 0\\ 0 & 0 & 0 \end{array}\\ \begin{array}{ccc} 0 & 0 & 0\\ 0 & 0 & 0\\ 0 & 0 & 0 \end{array} & \begin{array}{ccc} 1 & 0 & 0\\ 0 & 1 & 0\\ 0 & 0 & 1 \end{array} & \begin{array}{ccc} 0 & 0 & 0\\ 0 & 0 & 0\\ 0 & 0 & 0 \end{array}\\ \begin{array}{ccc} 0 & 0 & 0\\ 0 & 0 & 0\\ 0 & 0 & 0 \end{array} & \begin{array}{ccc} 0 & 0 & 0\\ 0 & 0 & 0\\ 0 & 0 & 0 \end{array} & \begin{array}{ccc} 1 & 0 & 0\\ 0 & 1 & 0\\ 0 & 0 & 1 \end{array} \end{array}\right],$$

(7) $$\left\{\dot{\varepsilon }\right\}=\left\{\begin{array}{c} {\dot{\varepsilon }}_{xx}\\ {\dot{\varepsilon }}_{yy}\\ {\dot{\varepsilon }}_{zz}\\ {\dot{\varepsilon }}_{xy}\\ {\dot{\varepsilon }}_{yz}\\ {\dot{\varepsilon }}_{zx} \end{array}\right\},,$$

where ${\dot{\varepsilon }}_{ij}$ is the strain rate tensor, and i,j∈x|y|z.

**Figure 9:**
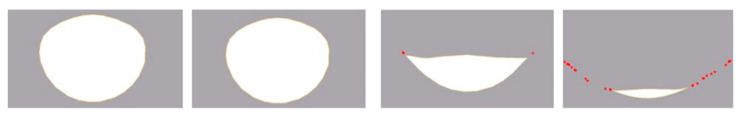
The main stages of pore healing under the action of a compressive force initiated by a laser pulse/plasma. The red dots show isolated healed areas. Modeling of the healing process was carried out for the pore closest to the surface of the uniformly heated material.

The result of modeling the individual stages of pore healing is shown in Figure 9.

In general, the distribution of the isotherms and the values of ΔT_1_ significantly depend on the location of the pores and the distance to the surface of the sample. It is evident from our modeling that the more elevated the temperature of the material and the value of ΔT_1_ are, the more intensive the process of pore healing is. In addition, for the upper pore, the direction of propagation of the isotherms coincides with the direction of the impact of the compression force. The isotherms are symmetrical with respect to this direction.

The simulation results obtained in this work allow us to describe the physical mechanism of pore healing, as well as the need for irradiation using a series of laser impulses. The influence of the first laser impulses stimulates the selective healing of the pores located close to the surface. The influence of subsequent laser impulses leads to healing of the pores located deeper. However, the effectiveness of pore healing significantly decreased with increasing distance from the surface. This is due to both a decrease in the temperature increment above the surface and a decrease in the shock pressure.

It is also noteworthy that the movement of isotherms depends on the relative arrangement of the pores. The results obtained in this work allow us to consistently explain the experimental data [2,23] and to propose a physical model for the selective laser healing of nano- and micropores on the surface layer of a metal alloy.

## 5. Conclusions

A physical mechanism for employing the selective effect of laser impulses with a duration of about 20 ns and an energy of 15–20 MJ on nanopores located on a surface of titanium alloy layer was proposed. The selective effect was provided by additional heating of the material above the pore and subsequent healing of the pore due to the movement of the heated material into the pore volume under the action of shock pressure.

The increase in nanohardness of the laser-treated surface by one and a half times or more was accompanied by an increasing resistance to cracking. For the untreated surface of a titanium alloy VT18u, the mathematical expectation of the probability of crack formation under the condition of local loading using a Vickers pyramid was 0.2 at F = 0.49 N and 0.55 at F = 4.9 N. There was no surface cracking on the laser-treated surface after local loading using a Vickers pyramid in the range loads of 0.49 N and up to 4.9 N inclusive.

The physical phenomenon underlying selectively laser-initiated pore healing at the nanoscale is determined by the specific distribution of isotherms and the shock compression of the material, which is influenced by both laser heating and the configuration of the nanopore system.

The proposed physical model consistently explains the experimental results associated with changes in the mechanical properties of the thin surface layer of the sample, in which, as a result of exposure to laser irradiation and laser plasma, a short-time transfer of the material to an extreme state is initiated.

## Figures and Tables

**Table 1:** Technical characteristics of the laser LS-2136.

Parameters	Value	Unit
Wavelength	532	nm
Energy	75	mJ
Pulse duration (FWHM)	15–20	ns
Pulse repetition rate	50	Hz
Beam divergence (Θ _0.86_)	≤0.7	mrad

**Table 2:** Microhardness HV and mathematical expectation of the probability of crack formation after loading using Vickers pyramid (P_me_) on titanium alloy VT18u before and after treatment with a series of laser pulses.

F, N	Untreated Surface, P_me_	Laser-Treated Surface, P_me_	Untreated Surface, HV	Laser-Treated Surface, HV
0.49 ± 0.00735	0.2	0	218 ± 3.27	308 ± 4.62
0.98 ± 0.0147	0.25	0	250 ± 3.75	355 ± 5.325
1.47 ± 0.02205	0.3	0	265 ± 3.975	360 ± 5.4
1.96 ± 0.0294	0.35	0	270 ± 4.05	355 ± 5.325
2.94 ± 0.0441	0.4	0	(260–290) ± 4.125	(320–380) ± 5.25
3.92 ± 0.0588	0.45	0		
4.9 ± 0.0735	0.55	0		

## Data Availability

Data are contained within the article.
